# Herpes Simplex Virus 1 Tegument Protein UL46 Inhibits TANK-Binding Kinase 1-Mediated Signaling

**DOI:** 10.1128/mBio.00919-19

**Published:** 2019-05-21

**Authors:** Hongjuan You, Sisilia Zheng, Zhiming Huang, Yingying Lin, Qingtang Shen, Chunfu Zheng

**Affiliations:** a Department of Immunology, School of Basic Medical Sciences, Fujian Medical University, Fuzhou, China; b Jiangsu Key Laboratory of Immunity and Metabolism, Department of Pathogenic Biology and Immunology, Xuzhou Medical University, Xuzhou, Jiangsu, China; c Department of Psychology, University of Calgary, Calgary, Alberta, Canada; d Department of Microbiology, Immunology and Infectious Diseases, University of Calgary, Calgary, Alberta, Canada; University of California, Irvine

**Keywords:** HSV-1, IRF3, TBK1, UL46, type I IFN

## Abstract

TANK-binding kinase 1 (TBK1) is a key component of the antiviral immunity signaling pathway. It activates downstream interferon regulatory factor 3 (IRF3) and subsequent type I interferon (IFN-I) production. Herpes simplex virus type 1 (HSV-1) can antagonize host antiviral immune responses and lead to latent infection. Here, HSV-1 tegument protein UL46 was demonstrated to downregulate TBK1-dependent antiviral innate immunity. UL46 interacted with TBK1 and reduced TBK1 activation and its downstream signaling. Our results showed that UL46 impaired the interaction of TBK1 and IRF3 and downregulated the activation of IRF3 by inhibiting the dimerization of TBK1 to reduce the IFN-I production induced by TBK1 and immunostimulatory DNA. The IFN-I and its downstream antiviral genes induced by UL46-deficient HSV-1 (ΔUL46 HSV-1) were higher than those of wild-type HSV-1 (WT HSV-1). In addition, the stable knockdown of TBK1 facilitated the replication of ΔUL46 HSV-1, but not WT HSV-1. Together, these findings reveal a novel mechanism of immune evasion by HSV-1.

## Abstract (precis)

HSV-1 has evolved multiple strategies to evade host antiviral responses and establish a lifelong latent infection, but the molecular mechanisms by which HSV-1 interrupts antiviral innate immunity are not completely understood. As TBK1 is very critical for antiviral innate immunity, it is of great interest to reveal the immune evasion mechanism of HSV-1 by targeting TBK1. In the present study, HSV-1 UL46 was found to inhibit the activation of IFN-I by targeting TBK1, suggesting that the evasion of TBK1 mediated antiviral innate immunity by HSV-1 UL46. Findings in this study will provide new insights into the host-virus interaction and help develop new approaches against HSV-1 infection.

## INTRODUCTION

Viral infection triggers the innate immune response and subsequent production of type I interferon (IFN-I) and other antiviral factors, which constitute the first line of defense against virus invasion. The pathogen recognition receptors (PRRs) detect structurally conserved pathogen-associated molecular patterns (PAMPs) generated during the pathogen life cycle, which then activates the IFN-I signaling pathway cascade (1, 2). For DNA virus infection, cytosolic DNA sensors, such as cyclic GMP-AMP synthase (cGAS), bind to aberrant double-stranded DNA (dsDNA) and produce cyclic GMP-AMP. The latter, an important secondary messenger in the cytoplasm, activates stimulator of interferon gene (STING) and then recruits TANK-binding kinase 1 (TBK1), which leads to TBK1 dimerization and phosphorylation. These cascade reactions promote TBK1 kinase activity and phosphorylate interferon regulatory factor 3 (IRF3), which then leads to IRF3 nuclear localization and IFN-I production (3–5). Therefore, TBK1 is a key adaptor protein for antiviral immunity signaling pathway. It is constitutively expressed, and IRF3 activation is attenuated in TBK1-deficient cells (6).

Herpes simplex virus 1 (HSV-1), a number of the alphaherpesvirus subfamily, is a large linear double-stranded DNA virus that encodes more than 80 proteins. HSV-1 has the capacity to counteract host antiviral innate immune response by its viral proteins and establish a lifelong latent infection (7). HSV-1 tegument protein UL46 is a tyrosine-phosphorylated protein that accumulates late in viral infection. Despite its abundance, UL46 is required for the benefit of the virus even though it is not essential for viral replication in cells. During infection, UL46 is delivered into the cytoplasm as multiple punctate structures, and intracellular UL46 associates with both cellular membranes and viral capsids (8, 9). Previous studies proposed that when UL46 is bound to VP16, the promotion of the VP16-dependent transcriptional induction of genes (10) occurs. UL46 interacts with the lymphocyte-specific Src family kinase (SFK) Lck or Lck signaling complexes and activates Lck during HSV-1 infection. UL46 can be strongly tyrosine phosphorylated in lymphocytes depending on the activity of Lck for the benefit of the virus. The SFK-dependent phosphorylation of UL46 was required for UL46-phosphatidylinositol 3-kinase (PI3K) interactions and the activation of AKT during HSV-1 infection to modulate cellular signaling pathways (11–13). In addition, it is also important to reveal the role of UL46 in immune evasion of HSV-1. Recent studies showed that UL46 interacted with STING and eliminated the transcripts of both STING and IFI16 to suppress innate immune responses and became beneficial for HSV-1 infection (14). However, the underlying mechanism by which UL46 counteracts the host antiviral innate immune response is still elusive.

In the present study, we identified UL46 as a negative regulator of antiviral innate immunity by binding to TBK1. This impairs TBK1 activation and suppresses TBK1-mediated IFN-I signaling. We found that UL46 reduced the dimerization of TBK1 and impaired the interaction between TBK1 and IRF3 to inhibit the activation of IRF3. Additionally, our study demonstrated that the IFN-β and its downstream IFN-stimulated genes (ISGs) induced by UL46-deficient HSV-1 (ΔUL46 HSV-1) were higher than those of wild-type HSV-1 (WT HSV-1). UL46 inhibited activation of the IFN-I signaling pathway which was induced by immunostimulatory DNA (ISD). In addition, stable knockdown of TBK1 facilitated the replication of ΔUL46 HSV-1, but not WT HSV-1. In summary, these findings provide new insights into the host-virus interaction and reveal a novel mechanism of immune evasion by HSV-1.

## RESULTS

### UL46 inhibits IFN-β signaling pathway stimulated by TBK1.

**FIG 1:**
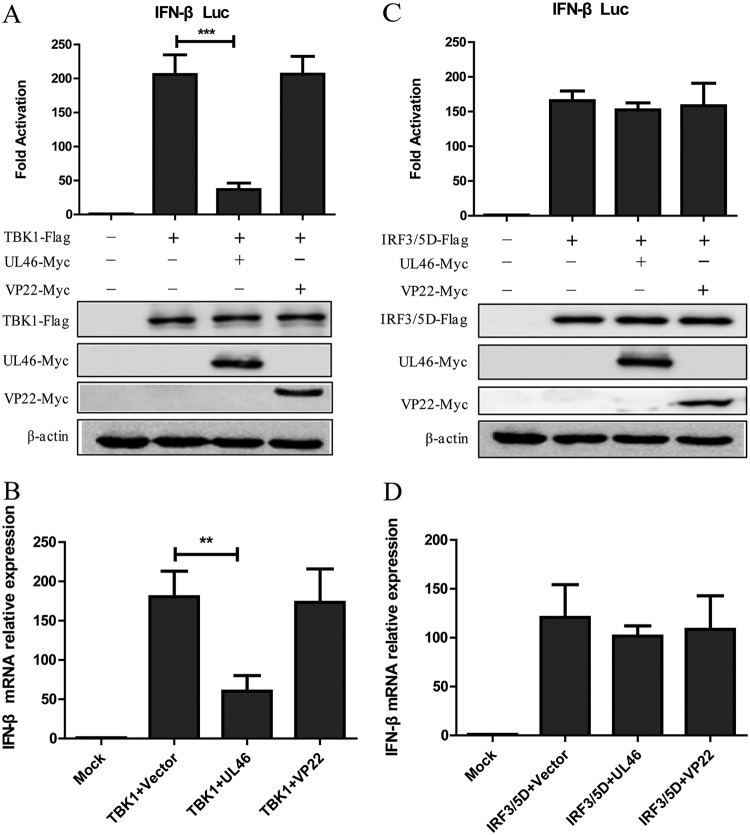
UL46 inhibits the IFN-β signaling pathway induced by TBK1. (A and C) HEK293T cells were transfected with IFN-β-Luc reporter, pRL-TK and TBK1-Flag (A), IRF3/5D-Flag (C) along with vector, UL46-Myc or VP22-Myc plasmid. Cells were harvested at 24 h after transfection and subjected to DLR assay. The expression of TBK1, IRF3/5D, and UL46 were analyzed by WB using anti-Flag, anti-Myc, and anti-β-actin (as a control) MAbs. (B and D) HEK293T cells were transfected with TBK1-Flag (B) or IRF3/5D-Flag (D) along with vector, UL46-Myc or VP22-Myc plasmid for 24 h, and the cells were harvested and subjected to qRT-PCR analysis. The data represent results from one of the triplicate experiments. Error bars represent SDs from three independent experiments. Statistical analysis was performed using Student’s *t* test with GraphPad Prism 5.0 software. Values that are significantly different are indicated by bars and asterisks as follows: **, 0.001*<P < *0.01; ***, *P < *0.001.

To evaluate whether individual viral proteins could regulate the TBK1-mediated IFN-β pathway, HEK293T cells were cotransfected with IFN-β-Luc reporter, pRL-TK and TBK1 expression plasmid, along with vector plasmid or HSV-1 protein plasmids to screen for viral proteins that could inhibit IFN-β promoter activation by dual-luciferase reporter (DLR) assays. We found that ectopic expression of TBK1 activated the IFN-β promoter in HEK293T cells, and cotransfection of UL46, but not VP22, which has been reported to inhibit the production of IFN-β by targeting cGAS (15), significantly inhibited the activation of the IFN-β promoter (Fig. 1A). Further studies showed that ectopic expression of UL46 did not inhibit activation of the IFN-β promoter driven by the active form of IRF3 (IRF3/5D), which consists of 5-amino-acid-residue mutation in the C-terminal domain of IRF3 and leads to its nuclear translocation and activates IFN-β promoter directly (Fig. 1C). Also, as shown in Fig. 1B and D, UL46 inhibited the accumulation of *IFN-β* mRNA induced by TBK1, but not IRF3/5D. Collectively, these results suggest that UL46 specifically inhibits the IFN-β production induced by TBK1.

### HSV-1 inhibits IFN-β activation by UL46.

**FIG 2:**
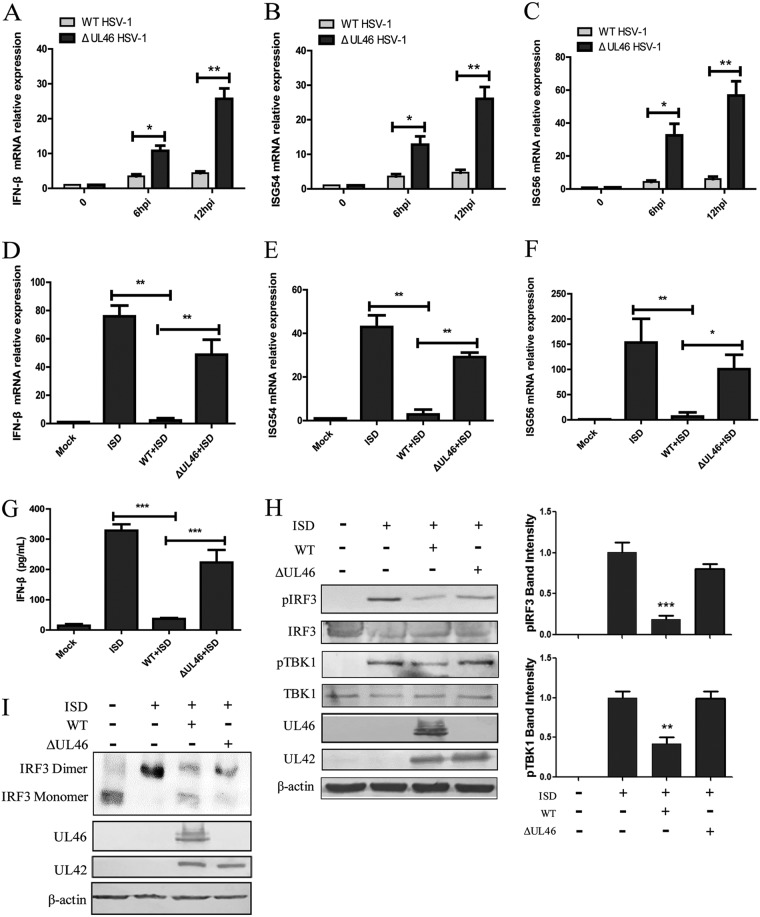
HSV-1 downregulates the production of both IFN-β and ISGs via UL46. (A to C) HFF cells were infected with WT HSV-1 or ΔUL46 HSV-1 at an MOI of 5 for the indicated times (hpi, hours postinfection), and total RNA was extracted and used for quantification of the IFN-β (A), ISG54 (B), and ISG56 (C) by qRT-PCR. (D to G) HFF cells were infected with WT HSV-1 or ΔUL46 HSV-1 (MOI = 5) for 2 h, then the cell medium was replaced, and cells were transfected with ISD (2 μg/ml) using Lipofectamine LTX. (D to G) Cells were harvested and subjected to qRT-PCR analysis at 7 h posttransfection (D to F) or ELISA analysis (G) at 18 h posttransfection. (H and I) HFF cells were infected and transfected as described for panel D, and then the cells were harvested at 7 h posttransfection and subjected to WB analysis to detect the phosphorylation of TBK1 (pTBK1) and IRF3 (pIRF3) (H) and native PAGE to detect IRF3 dimerization (I). The levels of pTBK1 and pIRF3 were quantified using densitometry analysis. The data represent results from one of the triplicate experiments. Statistical analysis was performed using Student’s *t* test with GraphPad Prism 5.0 software. Statistical significance symbols: *, 0.01*<P < *0.05; **, 0.001*<P < *0.01; ***, *P < *0.001.

To investigate the function of UL46 on the IFN-β signal pathway in the context of HSV-1 infection, we examined the expression of IFN-β and its downstream ISGs in human foreskin fibroblast (HFF) cells infected with WT HSV-1 or ΔUL46 HSV-1, and quantitative reverse transcription-PCR (qRT-PCR) was performed to measure *IFN-β*, *ISG54*, and *ISG56* mRNA accumulation. As shown in Fig. 2A, the IFN-β production induced by ΔUL46 HSV-1 was significantly higher than that of WT HSV-1 in HFF cells from 6 to 12 h, and similar results were found for ISG54 and ISG56 (Fig. 2B and C). These data indicated that UL46 downregulated the activation of the IFN-I signaling pathway. To further confirm the role of UL46 in immune evasion by HSV-1, HFF cells were infected with WT HSV-1 or ΔUL46 HSV-1 for 2 h and then transfected with immunostimulatory DNA (ISD), a double-stranded DNA 60-mer oligonucleotide derived from the HSV-1 genome with a high capacity to induce IFN-β production. Cells were also harvested and subjected to qRT-PCR to analyze *IFN-β*, *ISG54*, and *ISG56* mRNA. As shown in Fig. 2D to F, infection of HFF cells with WT HSV-1, but not ΔUL46 HSV-1, significantly inhibited the accumulation of *IFN-β*, *ISG54*, and *ISG56* mRNA induced by ISD, suggesting that HSV-1 UL46 inhibited the production of IFN-β induced by ISD. To confirm this assertion, ELISAs were performed to measure the secretion of IFN-β in the supernatant of HFF cells infected by WT HSV-1 or ΔUL46 HSV-1 followed by the transfection with ISD. IFN-β was significantly decreased in cells infected with WT HSV-1, while ΔUL46 HSV-1 infection recovered IFN-β production to a certain extent (Fig. 2G).

In the IFN-I signal pathway, IRF3 is a key transcription factor activated by TBK1. After the activation of the IFN-I signal pathway, TBK1 is phosphorylated and then activates IRF3 by phosphorylation and dimerization of the latter to stimulate IFN-I production. These are hallmarks of early antiviral responses mediated by IFN-I. To confirm the inhibitory effect of UL46 on the IFN-I signal pathway, the phosphorylation of TBK1 and IRF3 was detected in HFF cells during viral infection. As shown in Fig. 2H, ISD alone induced robust phosphorylation of TBK1 and IRF3. However, infection with WT HSV-1, but not ΔUL46 HSV-1, inhibited the phosphorylation of TBK1 and IRF3 induced by ISD, which is critical for IFN-I production. We also found that infection with WT HSV-1 inhibited the dimerization of IRF3 induced by ISD, while infection with ΔUL46 HSV-1 resumed dimerization of IRF3 to a certain extent (Fig. 2I). Collectively, these results indicated that UL46 plays an important role in immune evasion by HSV-1.

### UL46 associates with TBK1 and inhibits activation of TBK1.

**FIG 3:**
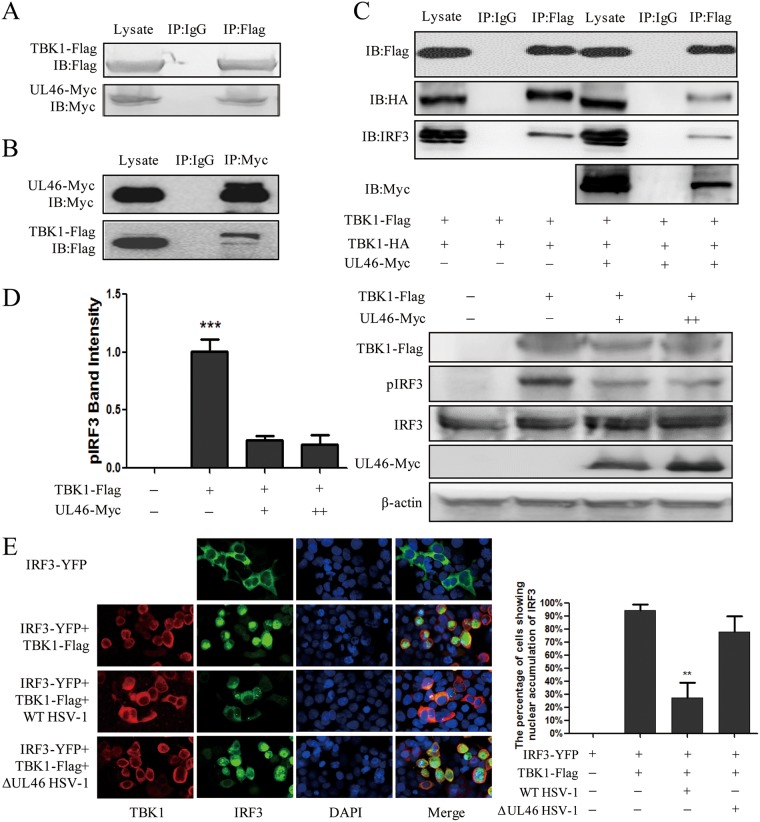
UL46 associates with TBK1 and inhibits the activation of TBK1. (A and B) HEK293T cells were transfected with TBK1-Flag and UL46-Myc for 36 h. The cells were lysed, and the extracts were subjected to IP with anti-Flag MAb (A), anti-Myc MAb (B), or control IgG. Precipitates were analyzed by WB. (C) HEK293T cells were transfected with both TBK1-Flag and TBK1-HA along with UL46-Myc or vector plasmid. The cells were harvested at 36 h posttransfection and subjected to IP with anti-Flag MAb or control IgG. Precipitates were analyzed by WB. IB, immunoblotting. (D) HEK293T cells were transfected with TBK1-Flag along with UL46-Myc or vector plasmid for 24 h. Cells were harvested and subjected to WB analysis to detect IRF3 phosphorylation (pIRF3), and the levels of pIRF3 were quantified using densitometry analysis. (E) HEK293T cells were infected with WT HSV-1 or ΔUL46 HSV-1 for 2 h and then transfected with IRF3-YFP along with vector or TBK1-Flag for 24 h. Cells were stained with mouse anti-Flag MAb, and TRITC-conjugated goat anti-mouse (red) antibodies were used as the secondary antibodies. Cell nuclei (blue) were stained with Hoechst 33258. The images were obtained by confocal microscopy. The data represent results from one of the triplicate experiments. Error bars represent SDs from three independent experiments. Statistical analysis was performed using Student’s *t* test with GraphPad Prism 5.0 software. **, 0.001*<P < *0.01; ***, *P < *0.001.

Previous studies showed that homodimerization and phosphorylation of TBK1 are required for its activation (16–18). The activated TBK1 phosphorylates IRF3 and leads to the latter nuclear translocation and activation by binding to IRF3 (1, 2, 19). To clarify the underlying molecular mechanism by which UL46 inhibited the IFN-I signal pathway, a coimmunoprecipitation (co-IP) assay was employed to investigate the interaction between UL46 and TBK1 and the interaction of TBK1 with TBK1 or IRF3 in the presence or absence of ectopic expression of UL46. As shown in Fig. 3A and B, UL46 associated with TBK1. Overexpression of exogenous TBK1 would promote its self-association and autoactivation. Then the activated TBK1 initiates IRF3 activation to induce IFN-β expression by combination with IRF3. Interestingly, the association of UL46 with TBK1 inhibited the homodimerization of TBK1 and the interaction between TBK1 and IRF3 (Fig. 3C). These results suggested that UL46 directly inhibited TBK1 activation. Furthermore, ectopic expression of UL46 distinctly inhibited the phosphorylation of IRF3 stimulated by TBK1 (Fig. 3D). Finally, the immunofluorescence assay was performed in HEK293T cells transfected with IRF3-YFP along with vector plasmid or TBK1 expression plasmid following WT HSV-1 or ΔUL46 HSV-1 infection. As shown in Fig. 3E, IRF3 remained in the cytoplasm when expressed alone and ectopic expression of TBK1 induced nuclear translocation of IRF3 in 95% of cells. WT HSV-1 infection prevented the nuclear translocation of IRF3 induced by TBK1 in more than 65% of cells. However, ΔUL46 HSV-1 resumed the nuclear translocation of IRF3 in about 80% of cells. Taken altogether, these results indicated that UL46 prevented IFN-β activation by inhibiting TBK1 activation.

### TBK1 mediates the defense against the replication of ΔUL46 HSV-1.

**FIG 4:**
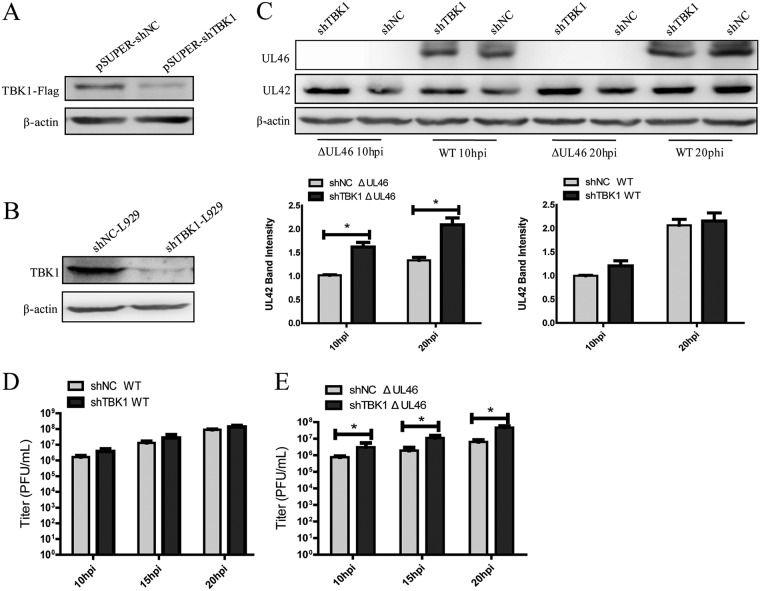
TBK1 mediates the defense against the replication of ΔUL46 HSV-1. (A) HEK293T cells were transfected with TBK1-Flag and pSUPER-shNC or the indicated pSUPER-shTBK1 plasmids, and cells were harvested at 72 h posttransfection and subjected to WB analysis. (B) WB analysis of endogenous TBK1 using cell lysates of L929-shNC and L929-shTBK1 cells. (C to E) The stably transfected L929-shNC and L929-shTBK1 cells were infected with WT HSV-1 or ΔUL46 HSV-1 at an MOI of 1, then harvested at the indicated time points postinfection, and subjected to WB analysis (C) or viral plaque assay on Vero cells (D and E). The data represent results from one of the triplicate experiments. Statistical analysis was performed using Student’s *t* test with the GraphPad Prism 5.0 software. *, 0.01*<P < *0.05.

Previous studies have revealed that the activation of TBK1 elicits a potent antiviral response, while the aforementioned data suggested that HSV-1 infection inhibited activation of the IFN-β pathway by targeting TBK1. To better delineate the antiviral role of TBK1 on HSV-1 replication, the stably transfected L929-shNC (NC stands for negative control) and L929-shTBK1 cells were constructed. Western blot (WB) analysis was performed to evaluate the knockdown efficiency of TBK1 RNA interference (RNAi) plasmid. HEK293T cells were transfected with TBK1-Flag and pSUPER-shNC or pSUPER-shTBK1 plasmids, and then cells were harvested and subjected to WB analysis (Fig. 4A). L929 cells were transfected with pSUPER-shTBK1 or pSUPER-shNC plasmid to screen for stably transfected cell lines. As shown in Fig. 4B, the expression of endogenous TBK1 in L929-shTBK1 cells was markedly decreased compared to that in L929-shNC cells. Then, the stably transfected L929-shNC and L929-shTBK1 cells were infected with WT HSV-1 or ΔUL46 HSV-1 and harvested at the indicated time points for WB analysis and viral plaque assay. The levels of expression of UL42 in L929-shNC and L929-shTBK1 cells were similar upon WT HSV-1 infection, while protein levels of UL42 were increased in L929-shTBK1 cells compared to those in L929-shNC cells upon ΔUL46 HSV-1 infection (Fig. 4C). Similarly, viral plaque assay also demonstrated that knockdown of TBK1 did not affect the replication of WT HSV-1 but facilitated the replication of ΔUL46 HSV-1 (Fig. 4D and E). Collectively, these data indicated that TBK1 mediated the defense against the replication of ΔUL46 HSV-1.

## DISCUSSION

The innate immune system is the first line of host defense against invading pathogens, and IFN-I is particularly important for early control of viral infection. To successfully establish and maintain infection, HSV-1 has the ability to evade host antiviral machinery and facilitate viral infection and replication. Previous studies showed that HSV-1 proteins such as US3, ICP0, VP16, UL42, UL24, VP22, UL36USP, US11, and UL41 can inhibit IFN-I production and NF-κB activation by targeting IRF3, p65, IκBα, or cGAS (15, 20–29). In addition, HSV-1 inhibits the expression of ISGs to facilitate its own replication in host cells (30–33). To further reveal the underlying molecular mechanism of immune evasion by HSV-1, we used WT HSV-1 and HSV-1 with specific genes deleted to identify UL46 as a critical factor to downregulate the IFN-I signaling pathway.

During HSV-1 infection, both RNA and DNA of viruses would stimulate IFN-I production. TBK1 sits at the center of antiviral innate immune responses to both the DNA and RNA sensing pathways, so the screening of HSV-1 proteins to inhibit activation of IFN-I by TBK1 would in turn deepen our understanding of the underlying immune evasion mechanisms by HSV-1. Previous studies showed that ICP34.5 formed complexes with TBK1 and prevented production of interferon and ISGs (34), and ICP27 interacted with TBK1 and STING, which then inhibited IFN-I production that is dependent on the RGG motif of ICP27 (35). A recent study showed that US11 disrupted the HSP90-TBK1 complex by binding to HSP90 to inhibit antiviral immunity (36). To further refine our understanding of the underlying molecular mechanisms of HSV-1 by targeting TBK1, a screening assay was performed to identify viral proteins that could inhibit IFN-β promoter activation triggered by TBK1, and HSV-1 tegument protein UL46 was shown to inhibit TBK1-mediated IFN-I production.

In this study, ectopic expression of UL46 was shown to significantly affect the production of IFN-β and ISGs induced by TBK1, but not IRF3/5D, indicating that UL46 inhibited the IFN-I signaling pathway at TBK1 or upstream of TBK1. VP22 was demonstrated to interact with cGAS and inhibit the enzymatic activity of cGAS to downregulate the IFN-β production (15). Therefore, VP22 was used as a negative control to confirm that UL46 specifically inhibited TBK1-mediated IFN-β activation. Then viral infection was used to verify the role of UL46 in IFN-β production. Infection with ΔUL46 HSV-1, but not WT HSV-1, resumed the production of IFN-β. Moreover, infection with WT HSV-1, but not ΔUL46 HSV-1, inhibited IFN-β production induced by ISD. Then further studies revealed that UL46 disrupted the self-dimerization of TBK1 and the subsequent phosphorylation of IRF3 via interaction with TBK1, leading to reduction of IRF3 nuclear transport induced by TBK1. Moreover, the stable knockdown of TBK1 significantly facilitated the replication of ΔUL46 HSV-1 but not WT HSV-1. These results further confirmed the contribution of immune evasion by UL46. A recent report showed that constitutively expressing UL46 eliminated the accumulation of transcripts of both STING and IFI16 to block innate immunity, while the protein level of STING remained stable throughout infection with the wild-type virus or the ΔUL46 virus (14). Our results demonstrated that the level of STING protein was not affected during both WT HSV-1 and ΔUL46 HSV-1 infection, which was consistent with previous reports (14). We found that UL46 interacted with TBK1. However, we did not find that UL46 promoted STING degradation. In our studies, transient transfection, but not stable transfection, was employed for ectopic expression of UL46, and the expression of STING was not affected by UL46 (data not shown). Under this circumstance, the potential role of UL46 on STING was excluded. UL46 was shown to directly inhibit the activation of TBK1, downstream of STING. Such a role of UL46 in IFN-I production was more direct and more effective, and a novel immune evasion mechanism by UL46 was demonstrated during viral infection.

In summary, HSV-1 tegument protein UL46 was shown here for the first time to counteract the activation of the IFN-I signal pathway by targeting TBK1. It inhibited the IFN-I production by UL46-TBK1 interaction, which affected the activity of TBK1 and eventually facilitated viral replication. Our findings in this study would be important for understanding the interaction between HSV-1 replication and host antiviral immune response.

## MATERIALS AND METHODS

### Cells, viruses, and antibodies.

HEK293T, HFF, L929, and Vero cell lines were obtained from the American Type Culture Collection (Manassas, VA) and cultured in Dulbecco’s modified Eagle medium (DMEM) (Gibco-BRL) supplemented with 10% fetal bovine serum (FBS) and 100 U/ml of penicillin and streptomycin. The WT HSV-1 KOS strain and ΔUL46 HSV-1 were propagated in Vero cells and titrated as previously described (13). The protease inhibitor mixture cocktail was purchased from Thermo Fisher Scientific (MA, USA). RIPA lysis buffer was purchased from Beyotime (Shanghai, China). Mouse anti-HA, anti-Myc, and anti-Flag monoclonal antibodies (MAbs) were purchased from Abmart (Shanghai, China). Mouse anti-β-actin MAb were purchased from Santa Cruz Biotechnology (Santa Cruz, CA). Rabbit anti-IRF3, anti-TBK1, anti-UL42, and anti-UL46 polyclonal antibodies (PAbs) were made by GL Biochem Ltd. (Shanghai, China). Phospho-IRF-3 (Ser396) and phospho-TBK1 were purchased from Cell Signaling Technology (Danvers, MA, USA).

### Plasmid construction.

All enzymes used for cloning procedures were purchased from Vazyme (Nanjing, China). UL46-Myc, pcDNA3.1-Flag-TBK1, pcDNA3.1-HA-TBK1, and IRF3-YFP expression plasmids were constructed by standard molecular biology techniques. Small hairpin RNA specific for TBK1 (shTBK1) and scrambled small hairpin RNA as a negative control (shNC) were cloned into pSUPER.retro.puro vector (Oligoengine, LA) to yield pSUPERshTBK1 and pSUPER-shNC plasmids, respectively, as described in our previous study (31). The shTBK1 primers were as follows: forward, 5′-GATCCCCTGCGTATGGACTTCCAGAATTCAAAGATTCTGGAAGTCCATA CGCATTTTTA-3′, and reverse, 5′-AGCTTAAAAATGCGTATGGACTTCCAGAATCTCTTGAATTCTGGAAGTCCATACGCAGGG-3′. A commercial reporter plasmid of pRL-TK was purchased from Promega Corporation (Madison, WI, USA). Other plasmids used include the following: pcDNA3.1-Flag-TBK1 (37), IRF3/5D (38), and IFN-β promoter reporter plasmid (39).

### Co-IP and Western blot analysis.

Co-IP assays and Western blot (WB) analysis were performed as previously described (40). Briefly, cells were transfected with plasmids as indicated in the figure legends. Harvested cells were lysed on ice with lysis buffer. The lysates were incubated with the antibodies referred to in the figure legends and protein A/G Plus-Agarose (Santa Cruz Biotechnology) overnight at 4°C. The beads were washed three times with lysis buffer, and WB analysis was performed as previously described to detect the interaction of proteins (28).

### RNA isolation and qRT-PCR.

Total RNA was extracted using TRIzol (Invitrogen, California) according to the manufacturer’s manual. Samples were dissolved with RNase-free water, digested with DNase I, and then subjected to reverse transcription as previously described. The cDNA was used as the template for qRT-PCR to detect the levels of IFN-β and ISGs, and 18S rRNA was used as an internal reference as previously described (32). The primers used in qPCR follow: for *IFN-β*, forward, 5′-CCAACAAGTGTCTCCTCCAAAT-3′, and reverse, 5′-AATCTCCTCAGGGATGTCAAAGT-3′; for *ISG54,* forward, 5′- CTGCAACCATGAGTGAGAA-3′, and reverse, 5′-CCTTTGAGGTGCTTTAGATAG-3′; for *ISG56,* forward 5′- TACAGCAACCATGAG TACAA-3′, and reverse, 5′-TCAGGTGTTTCACATAGGC-3′; for *18S rRNA*, forward 5′- CGGCTACCACATCCAAGGAA-3′, and reverse, 5′-GCTGGAATTACCGCGGCT-3′.

### Transfection and dual-luciferase reporter (DLR) assays.

HFF cells and L929 cells were transfected with Lipofectamine LTX (Invitrogen, CA, USA) according to the manufacturer’s recommendations. HEK293T cells were transfected with reporter plasmids, such as IFN-β-Luc and internal control plasmid pRL-TK, with or without expression plasmids, as indicated, by Lipofectamine 2000 (Invitrogen, CA, USA). At 24 h posttransfection, luciferase assays were performed with a dual-specific luciferase assay kit (Promega, Madison, WI) as described in our previous studies (24, 41).

### ELISA.

Concentrations of the IFN-β in cell culture supernatants were determined by VeriKine Human Interferon Beta ELISA kit from PBL Assay Science (Piscataway, NJ, USA), according to the manufacturer’s instructions.

### Native PAGE.

Native polyacrylamide gel electrophoresis (PAGE) was performed using ReadyGels (7.5%; Bio-Rad) as described in our previous study (23). Briefly, gels were prerun with 25 mM Tris and 192 mM glycine (pH 8.4) with 1% deoxycholate (DOC) in the cathode chamber for 30 min at 40 mA. Samples in native sample buffer (10 μg protein, 62.5 mM Tris-Cl [pH 6.8], 15% glycerol, and 1% DOC) were size fractionated by electrophoresis at 80 V and transferred to PVDF membranes for WB analysis.

### Immunofluorescence assays.

Immunofluorescence assays were performed as described previously (42). In brief, HEK293T cells were infected with WT HSV-1 or ΔUL46 HSV-1 for 2 h and then transfected with the IRF3-YFP along with TBK1-Flag or vector plasmid for 24 h and then fixed in 4% paraformaldehyde. Cells were incubated with mouse anti-Flag MAb (diluted 1:1000), followed by incubation with tetramethyl rhodamine isocyanate (TRITC)-conjugated goat anti-mouse IgG (Sigma-Aldrich). After each incubation step, cells were washed extensively with phosphate-buffered saline (PBS). Samples were analyzed by using a confocal microscope.

### Establishment of TBK1 stable knockdown L929 cells.

L929 cells were transfected with pSUPER-shTBK1 or pSUPER-shNC for 48 h, and then puromycin was added to the cells at a concentration of 1,000 ng/ml to screen the transfected cells. The stably transfected L929-shNC and L929-shTBK1 cells were then cultured with puromycin (500 ng/ml).

### Statistical analysis.

Data were represented as means ± standard deviations (SDs) when indicated, and Student’s *t* test was used for all statistical analyses with the GraphPad Prism 5.0 software. Differences between groups were considered significant when *P* value was *<*0.05.
