# Correction for You et al., “Herpes Simplex Virus 1 Tegument Protein UL46 Inhibits TANK-Binding Kinase 1-Mediated Signaling”

**DOI:** 10.1128/mbio.01693-23

**Published:** 2023-10-16

**Authors:** Hongjuan You, Sisilia Zheng, Zhiming Huang, Yingying Lin, Qingtang Shen, Chunfu Zheng

Volume 21, no. 3, e00919-19, 2019, https://doi.org/10.1128/mBio.00919-19. Fujian Medical University should not be listed as an affiliation for Hongjuan You and Chunfu Zheng.

